# Individual social capital and survival: a population study with 5-year follow-up

**DOI:** 10.1186/1471-2458-14-1025

**Published:** 2014-10-02

**Authors:** Linda Ejlskov, Rikke N Mortensen, Charlotte Overgaard, Line R B U Christensen, Henrik Vardinghus-Nielsen, Stella R J Kræmer, Mads Wissenberg, Steen M Hansen, Christian Torp-Pedersen, Claus D Hansen

**Affiliations:** Department of Health Science and Technology, Public Health and Epidemiology, Aalborg University, Niels Jernes Vej 14, 9220 Aalborg, Denmark; Department of Clinical Epidemiology, Aalborg University Hospital, Sdr. Skovvej 15, 9000 Aalborg, Denmark; Department of Sociology and Social Work, Aalborg University, Kroghstræde 7, 9220 Aalborg Øst, Denmark; Department of Cardiology, Copenhagen University Hospital, Gentofte, Niels Andersens Vej 65, 2900 Hellerup, Denmark

**Keywords:** Social capital, Mortality, Proportional hazards models, Gender differences, Trust, Social participation, Social environment, Expectations of reciprocity, Effect modifier

## Abstract

**Background:**

The concept of social capital has received increasing attention as a determinant of population survival, but its significance is uncertain. We examined the importance of social capital on survival in a population study while focusing on gender differences.

**Methods:**

We used data from a Danish regional health survey with a five-year follow-up period, 2007–2012 (n = 9288, 53.5% men, 46.5% women). We investigated the association between social capital and all-cause mortality, performing separate analyses on a composite measure as well as four specific dimensions of social capital while controlling for covariates. Analyses were performed with Cox proportional hazard models by which hazard ratios and 95% confidence intervals were calculated.

**Results:**

For women, higher levels of social capital were associated with lower all-cause mortality regardless of age, socioeconomic status, health, and health behaviour (HR = 0.586, 95% CI = 0.421-0.816) while no such association was found for men (HR = 0.949, 95% CI = 0.816-1.104). Analysing the specific dimensions of social capital, higher levels of trust and social network were significantly associated with lower all-cause mortality in women (HR = 0.827, 95% CI = 0.750-0.913 and HR = 0.832, 95% CI = 0.729-0.949, respectively). For men, strong social networks were associated with a higher risk of all-cause mortality (HR = 1.132, 95% CI = 1.017-1.260). Civic engagement had a similar effect for both men (HR = 0.848, 95% CI = 0.722-0.997) and women (HR = 0.848, 95% CI = 0.630-1.140).

**Conclusions:**

We found differential effects of social capital in men compared to women. The predictive effects on all-cause mortality of four specific dimensions of social capital varied. Gender stratified analysis and the use of multiple indicators to measure social capital are thus warranted in future research.

**Electronic supplementary material:**

The online version of this article (doi:10.1186/1471-2458-14-1025) contains supplementary material, which is available to authorized users.

## Background

Over the last two decades the concept of social capital has gained increasing popularity in public health and related discourses, making it one of the most popular used sociological concepts [1–3]. It has been argued that social capital is of profound importance for determining the effectiveness of community-based health promotion programmes [4] as well as a central element in the psychosocial explanation of health inequities [5, 6]. The concept is thus attaining a key role in the understanding of disparities in health and mortality rates [7, 8]. However, the cross-sectional nature of most studies that have investigated the relationship between social capital and health allows for limited causal interpretations [9].

It is generally agreed that social networks strengthen individuals and communities, and that the value can be seen as a form of embedded capital. Social networks thus influence people’s opportunities throughout their lives [2]. However, social capital is notoriously difficult to operationalize, as the widespread use of the concept across different disciplines has hampered consensus about its particulars [10, 11].

A recent review of the relationship between social capital and mortality has found that only one of 20 studies utilized a comprehensive definition of social capital while the remaining focused on one or only a few aspects of the concept [9]. A Spanish cohort study showed that higher social participation and stronger social networks had a positive effect on survival [12]. An Australian study showed that strong social networks were positively associated with higher survival. However this study solely included people aged 70 or older [13].

The dimension of trust plays a key role in social capital [11, 14, 15], and because of the limited investigation of this dimension the association between trust and all-cause mortality largely remains a grey area in research on social capital and health [9, 14]. However, studies that have examined the association indicated a positive relationship between trust and mortality moderated by gender. A Japanese study investigating the association between several different dimensions of social capital and mortality concluded that social networks were associated with all-cause mortality among older Japanese while mistrust was associated with lower mortality among women [16]. However, as the Japanese study pointed out, this is in a culturally different setting than the Western world. In a Western setting, a Finnish study also found a significant association between trust and lower all-cause mortality among women. For men, there was found a significant association between leisure participation and lower all-cause mortality [15].

Expectations of reciprocity is another important dimension that is rarely measured [14]. In this paper, a main focus is the analysis of four specific aspects of social capital including trust and expectations of reciprocity.

The definition of social capital is a critical issue, where views seem to depend on whether focus is on the sources or on the effects of social capital, and whether or not social capital should be sought at the group or individual level of society [1, 2, 17–19]. Social capital is often attributed to groups, whether in a residential community, at a work place, or in voluntary organizations [10]. Viewing social capital exclusively as a group attribute, however, can be problematic. According to Portes [2], social capital measured at this level may be seen both as a cause and an effect, and group level processes are often be mediated by individuals [20]. Our study views social capital as an individual attribute in alignment with the well-established research tradition in public health that has focused mainly on the positive effects of social relations on health [21]. But whereas the traditional research focuses on very specific areas of social relations i.e., social support and social ties social capital encapsulates several different aspects of social relationships at once as well as embedding important social relations in a more comprehensive theoretical framework [11]. In a sense, social support is a mechanism that links social capital with health outcomes (e.g. mortality) as was suggested by Kawachi et al. [10].

The gender issue is a key element in research on social capital [11]. Hyyppä et al. [15] found significant differences in statistical associations between the specific dimensions of social capital and mortality for the two genders: For women, higher levels of interpersonal trust were found to lower the risk of death, whereas this effect was significant only for men above 65 years of age. Skrabski et al. [22] established differential effects of leisure time activities for Hungarian men and women. According to Berkman [23] women are able to mobilize social support more effectively when compared to men, in addition to having access to more emotionally rewarding relationships. As a result, women’s way of relating to other people seems to be advantageous to their health [24]. On the other hand, Berkman found that the effect on their health may not always be positive [23], citing studies showing that women tend to become more involved when people in their network experience problems, and thus demand more of them. Despite the growing number of studies examining the association between social capital and health, the possible effects of gender remain unclear and associations are not fully understood [9]. Therefore, further analysis is needed.

In this study, we examined the association between individual-level social capital and all-cause mortality in a Danish follow-up study, paying special attention to gender differences. We employed a comprehensive operationalization of social capital and examined both a composite measure of social capital as well as its individual dimensions.

## Methods

### Study population and data sources

The study population was obtained from a Danish Regional Health Profile based on a 2007 survey entitled “How are you?”. The survey investigated factors relating to disease, quality of life, health behaviour, social capital and social relations. The survey was undertaken in 11 municipalities covering the entire Northern Denmark Region. A random selection of 23,490 people was approached, out of whom 11,497 persons aged 16–80 years (44.8% men and 55.2% women) responded to the postal questionnaire, representing 48.9% of the gross selection. These were followed until death or the 31^st^ of December 2012.

The nature of the publicly financed healthcare system in Denmark enables complete and nationwide registration on a variety of variables [25]. We obtained data on survival, annual income, and number and type of diagnoses from three registers. The Central Population Register (CPR) includes data on every person living in Denmark, with dates of birth, death, gender, etc. The Danish National Patient Register contains information on hospitalizations since 1978. The Danish Income Statistics Register holds data on income, taxes for Danish residents, etc. [25]. The Danish Data Protection Agency approved this retrospective register-based study (GEH-2014-014). An ethical approval is not required for a retrospective register-based study in Denmark.

### Mortality outcome

The mortality outcome variable was measured by data obtained from Central Population Register (CPR) [25].

### Social capital

**Figure 1:**
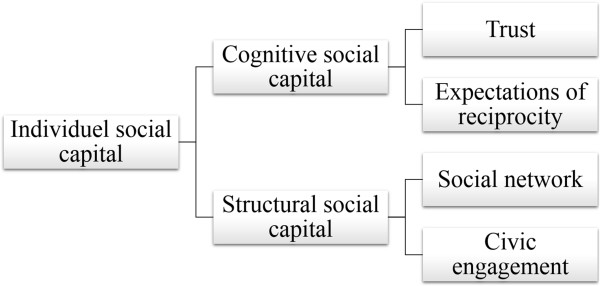
**Conceptualization of social capital.**

Following the operationalization used in several reviews and studies, we separated social capital into a cognitive and a structural component [4, 9, 16, 26, 27], which were subsequently divided into specific aspects in line with widely used definitions of individual social capital [11, 27] (see Figure 1).

Cognitive social capital is a measure of the individual’s level of trust towards others and perception of sharing and reciprocity while structural social capital reflects the density of the person’s social network and degree of civic engagement [11, 27]. Our paper investigates social capital both as a composite concept and along four specific dimensions. The responses to the dimensions listed in Table 1, interpersonal trust, expectations of reciprocity, participation in social networks, and civic engagement were used to measure social capital.

**Table 1:** **Questions underlying the social capital index**

Dimension	Question	Categories
Trust	Indicate your agreement with the following statement: “Most people can be trusted.”	1: completely disagree, 2: disagree 3: agree, 4: strongly agree, don’t know
	Indicate your agreement with the following statement: “Most people try to be fair most of the time.”	1: completely disagree, 2: disagree 3: agree, 4: strongly agree, don’t know
Expectations of reciprocity	Indicate your agreement with the following statement: “Most people would take advantage of you if they got the chance.”	1: completely disagree, 2: disagree 3: agree, 4: strongly agree, don’t know
	Indicate your agreement with the following statement: “You can’t be too careful when dealing with other people.”	1: completely disagree, 2: disagree 3: agree, 4: strongly agree, don’t know
Social network	“How often do you meet with friends that you don’t live with?”	1: never, 2; rarely, 3: once or twice a month 4: once or twice a week, 5: daily or almost daily, don’t know
	“How often do you meet with family that you don’t live with?”	1: never, 2; rarely, 3: once or twice a month 4: once or twice a week, 5: daily or almost daily, don’t know
Civic engagement	In your local community “How often do you participate in associations (for example committee work, evening classes, etc.)”	1:never, 2;rarely, 3: once or twice a month 4: once or twice a week, 5: daily or almost daily, don’t know
	“How often do you use the following facilities in your local community: church, religious activities, mosque, synagogue?”	1: never, 2; rarely, 3: once or twice a month 4: once or twice a week, 5: daily or almost daily, don’t know

Questions from the Danish Regional Health Profile, based on the 2007 survey “How are you?” (authors’ translation).

We chose to standardize the social capital variables due to the different scales of the social capital measures. The composite measure of social capital was created by summing the four standardized dimensions. Both the composite measure of social capital and the four specific dimensions are thus measured on a continuous scale.

### Covariates

We included in the models a number of independent variables which previous studies have shown as possible confounders [9, 15, 27]: age, gender, education, income, co-morbidity, self-rated health, living arrangement, tobacco use, alcohol consumption, and self-reported body mass index (BMI). Age was classified into four categories: 16–45, 46–59, 60–69, and above 70. Education was measured by the number of years in higher education, with 0 indicating no tertiary-level education. To obtain a stable picture, the household income variable was based on the average of 2005, 2006 and 2007 data as obtained from the Danish Income Statistics Register. Co-morbidity was measured by the Charlson index [28] via data from the Danish National Patient Register. Self-rated health was based on responses to the question “In general how do you assess your current health?” with five response categories ranging from very poor to very good*,* with don’t know responses coded as missing. Living Arrangement was categorized as a dichotomous variable indicating whether the respondents lived alone or not. Tobacco Use was measured as smoking at least once a week, rarely smokes, having quit smoking, or have never smoked. Alcohol Consumption was coded according to how often the respondents had consumed ≥ 5 units in the previous month: more than three times, about two to three times, one time, or not once. BMI was measured as below 19, between 19 and 25, between 25 and 30, or 30 or above.

### Statistical analysis

As the initial statistical analysis indicated that the association between social capital and all-cause mortality differed between the genders (p < 0.01, Table 2) regardless of age, socioeconomic status, health status, and health behaviour, we stratified all subsequent models by gender. Additionally, we also checked for an interaction with age but no reliable moderating effect was found. The statistical analyses were performed using the Cox proportional hazard models for the estimation of hazard ratio (HR) and 95% confidence intervals (95% CI) for all-cause mortality during the five-year follow-up period. Model A shows the estimates adjusted for age and gender while Model B further adjusts for socioeconomic status (education, living arrangements and income), health status (co-morbidity, self-rated health) and health behaviours (smoking, drinking, BMI). The estimates should be understood as when social capital increases by one standard deviation the survival rate changes by the hazard ratio. We used a design weight to correct for sample selection bias resulting from the sampling design. The questionnaire’s don’t know (DK) category (Table 1) was handled by directional coding [29]. To establish whether the DK responses would bias the results we performed a sensitivity analysis using three different methods: complete case analysis, directional coding, and multiple imputation, as suggested by Young and Kroh [29, 30]. Similar results were obtained across the three analyses, as shown in the Additional file 1. Only respondents with no missing on all of the independent variables were included in the final sample, resulting in 9,288 respondents (44.8% men, 55.2% women). Additionally, we have also performed an analysis that treated both the composite measure of social capital variable and the four specific dimensions as categorical variables with low, moderate and high levels of the corresponding variable. This analysis showed similar results to the analysis reported in this study.

**Table 2:** **Interaction effects between social capital, dimensions, and gender**

Variable	HR (95% CI)	Pr(>|z|)
Social capital^1^	1.6 (1.188-2.148)	< 0.01
Dimensions		
Trust^1^	1.21 (1.005-1.465)	< 0.05
Expectations of reciprocity^1^	1.06 (0.815-1.374)	0.669
Social network^1^	1.34 (1.192-1.496)	< 0.01
Civic engagement^1^	1.01 (0.790-1.297)	0.965

^1^Controlled for age, gender, socioeconomic status (education, living arrangements and income), health status (co-morbidity, self-rated health), and health behaviours (smoking, drinking, BMI).

HR (95% CI).

We performed the data management process using SAS software, version 9.4 (SAS institute Inc., Cary, North Carolina, USA) while all statistical analyses were performed using the R statistical software package, version 3.0.2 (R Development Core Team).

## Results

A total of 321 participants, 126 women and 195 men, died during the five-year follow-up period, corresponding to 3.5% of the respondents. Table 3 shows the other covariates and social capital variables according to gender.

**Figure 2:**
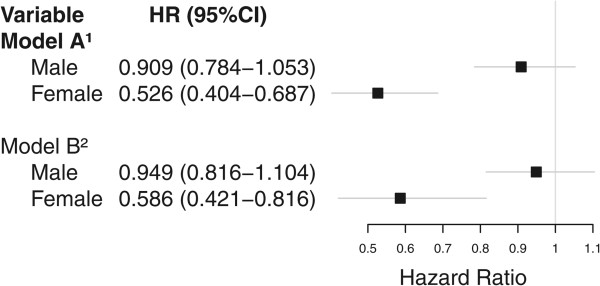
**Associations between social capital and all-cause mortality.** HR (95% CI). n = 9288. ^1^Includes age and gender. ^2^Includes age, gender, socioeconomic status (education, living arrangements and income), health status (co-morbidity, self-rated health), and health behaviours (smoking, drinking, BMI).

Figure 2 shows gender-specific associations between the composite social capital measure and all-cause mortality. No association was found between men’s social capital and mortality (HR = 0.909, 95% CI = 0.784-1.053), whereas for women, higher levels of social capital were significantly associated with a lower risk of all-cause mortality (HR = 0.526, 95% CI = 0.404-0.687). This association withstood control for socioeconomic status, age, health status, and health behaviour (HR = 0.586, 95%CI = 0.421-0.816).

**Table 3:** **Baseline characteristics, by gender**

Variable	Women n (%)	Men n (%)	p-value ^1^
Deceased	126 (2.5)	195 (4.5)	< 0.001
Social capital	0.04 {−0.29 , 0.40}^2^	0.00 {−0.36 , 0.33}^2^	< 0.001
Trust	0.08 {0.08 , 0.08}^2^	0.08 {0.08 , 0.08}^2^	0.68
Expectations of Reciprocity	0.12 {−0.67 , 0.91}^2^	0.12 {−0.67 , 0.12}^2^	< 0.001
Social networks	−0.17 {−0.90 , 0.56}^2^	−0.17 {−0.90 , 0.56}^2^	0.02
Civic engagement	0.04 {−0.39 , 0.48}^2^	0.04 {−0.39 , 0.48}^2^	0.00
Age			
16-45	1989 (40.0)	1449 (33.6)	
45-59	1577 (31.7)	1384 (32.1)	
60-70	1012 (20.4)	988 (22.9)	
>70	393 (7.9)	496 (11.5)	< 0.001
Co-morbidity			
No diseases	4898 (98.5)	4215 (97.6)	
One	32 (0.6)	61 (1.4)	
Two	25 (0.5)	29 (0.7)	
Three or more	16 (0.3)	12 (0.3)	0.00
Self-rated health			
Very good	874 (17.6)	717 (16.6)	
Good	2678 (53.9)	2362 (54.7)	
Neither good nor poor	1106 (22.2)	1005 (23.3)	
Poor	223 (4.5)	160 (3.7)	
Very poor	55 (1.1)	40 (0.9)	
Don’t know	35 (0.7)	33 (0.8)	0.23
Tertiery education in years	3 {1 , 4}^2^	4 {1 , 5}^2^	0.61
Household income			
Very low income	962 (19.4)	788 (18.3)	
Low income	1122 (22.6)	1012 (23.4)	
Average income	765 (15.4)	693 (16.1)	
Above-average income	960 (19.3)	783 (18.1)	
High income	872 (17.5)	780 (18.1)	
Very high income	290 (5.8)	261 (6.0)	0.4
Smoking			
At least once a week	1136 (22.9)	1073 (24.9)	
Rarely	127 (2.6)	115 (2.7)	
Has stopped	1156 (23.3)	1246 (28.9)	
Never smoked	2520 (50.7)	1862 (43.1)	
Don’t know	32 (0.6)	21 (0.5)	< 0.05
Living arrangement			
Live with partner	1044 (21.0)	826 (19.1)	
Live alone	3927 (79.0)	3491 (80.9)	0.03
Drinking (≥5 units of alcohol in last month)			
Never	3046 (61.3)	1736 (40.2)	
Once	1196 (24.1)	1276 (29.6)	
About 2–3 times	535 (10.8)	810 (18.8)	
About 4 times or more	143 (2.9)	436 (10.1)	
Don’t know	51 (1.0)	59 (1.4)	< 0.05
BMI			
Underweight (<19)	222 (4.5)	46 (1.1)	
Normal (19 > BMI < 25)	2714 (54.6)	1635 (37.9)	
Overweight (25 < BMI < 30)	1420 (28.6)	2025 (46.9)	
Obese (>30)	615 (12.4)	611 (14.2)	< 0.05

^1^χ^2^ or ANOVA test for gender differences.

^2^(median {1^st^ quartile, 3^rd^ quartile}).

**Figure 3:**
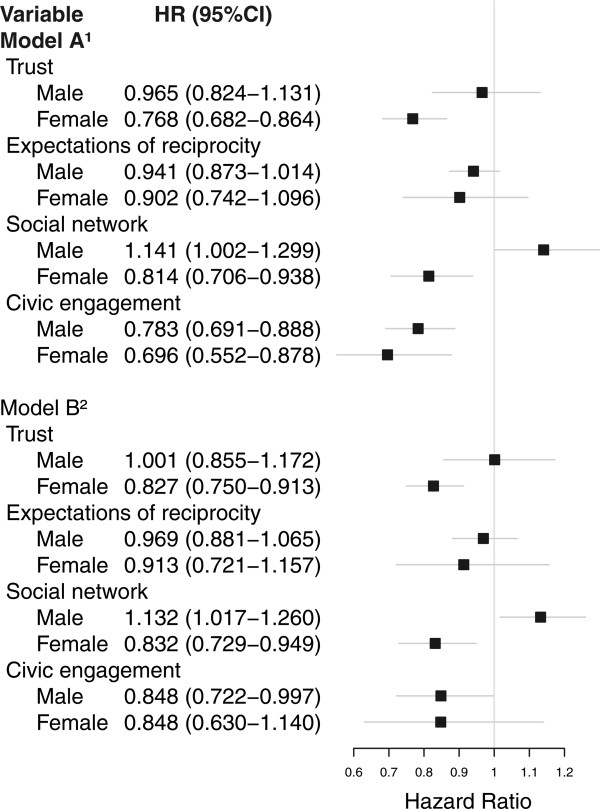
**Associations between social capital dimensions and all-cause mortality HR (95% CI).** n = 9288. ^1^Includes age and gender. ^2^Includes age, gender, socioeconomic status (education, living arrangements and income), health status (co-morbidity, self-rated health), and health behaviours (smoking, drinking, BMI).

Figure 3 shows associations between the four dimensions of social capital and all-cause mortality. We found clear indication of different predictive effects of trust and social networks for men and women (p < 0.05 and p < 0.01 respectively, Table 2). While higher levels of trust were significantly associated with lower all-cause mortality in women (HR = 0.768, 95% CI = 0.682-0.864), no evidence was found to support such an association in men (HR = 0.965, 95% CI = 0.824-1.131). A higher score on the social network dimension resulted in a lower all-cause mortality risk for women (HR = 0.814, 95% CI = 0.706-0.938) while men’s mortality risk was higher (HR = 1.141, 95% CI = 1.002-1.299). These results persisted when controlling for socioeconomic status, age, health status, and health behaviour. The results indicated no evidence of a different effect of civic engagement and expectation of reciprocity for men and women (p = 0.669 and p = 0.965, respectively; Table 2). No significant association was found between expectation of reciprocity and all-cause mortality; adjustment for confounders did not change this result. In the simple Model A adjusted only for age, higher civic engagement scores resulted in lower all-cause mortality risk for both men and women (HR = 0.783, 95% CI = 0.691-0.888 and HR = 0.696, 95% CI = 0.552-0.878). However, when controlling for socioeconomic status, health status, and health behaviour (Model B), the significant association between civic engagement and all-cause mortality disappeared for women (HR = 0.848, 95% CI = 0.60-1.140) while it remained significant for men (HR = 0.848, 95% CI = 0.722-0.997).

## Discussion

This paper has examined associations between social capital and all-cause mortality, and the modifying effect of gender. The results of our analyses show the differential effect of social capital on men and women, thus supporting earlier theoretical and empirical evidence [11, 15, 24, 31]. The study had two major findings, the first of which is that, when adjusted for age, socioeconomic status, health status, and health behaviour, stronger social capital is significantly associated with lower all-cause mortality in women while no significant association can be detected for men. Secondly, the results for the four studied dimensions of social capital differed in the strength of their association with all-cause mortality. Moreover, for two of the dimensions, men and women were differently affected.

As only a few studies examining social capital and mortality have included a comprehensive operationalization of social capital [9], this paper has concentrated on the analysis of four specific aspects of social capital. The analyses showed that expectations of reciprocity (unreliable results for both genders) and civic engagement have similar associations across gender, while the predictive effect of trust on mortality differed in the cognitive part, and network in the structural part. It thus seems that especially the trust and networks dimensions of social capital account for the differential predictive effect of social capital on all-cause mortality for men and women when age, socioeconomic status, health status, and health behavior are taken into account. These results emphasize that in research on health, several aspects of social capital should be taken into account. Concentrating on one aspect to the exclusion of all other may lead to biased results and invalid conclusions on the association between social capital and all-cause mortality, depending on which aspect is used to measure social capital.

Our results furthermore indicate that a strong social network, as measured by the frequency of contact with friends and family, increase men’s risk of dying, while this factor was associated with a lower risk of death in women. These results support the growing recognition that social capital can translate into both beneficial and detrimental effects on health [10, 31]. Several different theories may explain our finding that men with strong networks have higher all-cause mortality. Differences have already been established with regard to the nature of men’s and women’s social relationships [32]. Furthermore, a study have showed that men with higher social support appear to engage in both heavier drinking and have a higher fat intake than women [24]. Additionally, Hyyppä et al. [15] thus found their patterns of leisure participation to differ somewhat, with men participating in more risky activities compared to women. There are also studies indicating that women receive more support from their networks compared to men [33] and that they are more effective at mobilizing social support while also enjoying more emotionally rewarding relations [23]. This study thus supports the notion that there is a dark side of social capital [31] affecting male mortality negatively. Unfortunately, the questionnaire did not contain information about the specific types of activities carried out by the participants in relation to their social engagement. We were thus unable to examine which specific social engagements might be driving the negative association for men and help explain in more detail this ‘dark side of social capital. In the third installment of the study (collected in 2013) there are measures tapping into this which might prove helpful in enhancing our knowledge further. Regardless, further studies are needed for any distinct theory to be proposed.

High levels of trust proved predictive of a lower risk of all-cause mortality in women only. This supports previous findings from both Hyyppä et al. [15] and Aida et al. [16] thus giving cross-cultural indications that trust has a protective effect on mortality for women while none for men. Several possible explanations can be proposed; Giordano et al. suggest that high trust levels may reflect low levels of perceived social stress and anxiety [34]. Elstad offers the psychosocial explanation that trust in other people leads to lower stress and anxiety levels, which ultimately result in lower mortality [5]. This pathway has also been proposed by Abbott & Freeth [14], who argue that trust may act as protection against stress and anxiety by reducing apprehension about other people’s behaviour. Cacioppo et al. [35] suggest that feeling lonely is more damaging to a person’s health than actually spending comparatively long time alone. It could be argued that a feeling of loneliness reflects a person’s disinclination to trust other people, and that the two variables are intermingled in their effect on health. However, researchers of social capital and health have a rather vague understanding of the association between trust and all-cause mortality [9, 14], especially with regard to differences between men and women.

Besides the relatively large sample studied here [9], we count among the strengths of this study the operationalization of social capital as a multidimensional framework incorporating both cognitive and structural components. Moreover, data on mortality and several of the confounding variables were drawn from national registers, thus reducing the risk of differential misclassification and other sources of bias related to the measurement of these factors.

Some of the noteworthy limitations of this study stem from the use of a population sample drawn from a less urbanized region of Denmark, which calls for comparable investigations outside a Scandinavian context. Analysis of the non-response pattern revealed that there was a slight overrepresentation of women and older age groups in this study in line with studies of non-response patterns [36]. Research focused on substantive variables have concluded that response rates are unrelated to or only very weakly related to the distribution of substantive responses [36]. However, we cannot rule out a possible non-response bias attenuating the association between social capital and all-cause mortality thus affecting the validity of the results.

As mentioned above, there is no shared understanding of the operationalization of social capital in health research, which makes cross-study comparison challenging. Furthermore, we cannot claim that all relevant confounders have been controlled for although the longitudinal framework of our study strengthens a causal interpretation.

## Conclusion

This study shows that, regardless of socioeconomic status, health status, health behaviour, and age, the level of social capital is associated with all-cause mortality for women but not for men. The dimensions of trust and social network were important factors in the observed differential association of social capital across gender.

Our results emphasize the importance of stratifying for gender when performing analyses of social capital in health research. The different effects of the dimensions studied here indicate that using a single dimension to capture social capital may lead to biased results and invalid conclusions. We therefore recommend that the measurement of social capital is pursued along different lines and that future investigation includes the moderating effect of gender. We recommend further study to unravel the mechanisms underlying the observed differences in associations between social capital and all-cause mortality across gender.

## Electronic supplementary material

Additional file 1: **Results from sensitivity analysis – directional coding, complete data analysis, and multiple imputation.** ^1^ Includes age, gender, socioeconomic status (education, living arrangements and income), health status (co-morbidity, self-rated health), and health behaviours (smoking, drinking, BMI). (XLSX 13 KB)
